# Crosstalk between hypoxic cellular micro-environment and the immune system: a potential therapeutic target for infectious diseases

**DOI:** 10.3389/fimmu.2023.1224102

**Published:** 2023-07-31

**Authors:** Olalekan Chris Akinsulie, Sammuel Shahzad, Seto Charles Ogunleye, Ifeoluwa Peace Oladapo, Melina Joshi, Charles Egede Ugwu, Joy Olaoluwa Gbadegoye, Fasilat Oluwakemi Hassan, Richard Adeleke, Qudus Afolabi Akande, Ridwan Olamilekan Adesola

**Affiliations:** ^1^ Department of Veterinary Microbiology and Pathology, College of Veterinary Medicine, Washington State University, Pullman, WA, United States; ^2^ College of Veterinary Medicine, Mississippi State University, Starkville, MS, United States; ^3^ Center for Molecular Dynamics Nepal, Kathmandu, Nepal; ^4^ Paul G. Allen School for Global Health, Washington State University, Pullman, WA, United States; ^5^ Department of Physiology, University of Tennessee Health Science Center, Memphis, TN, United States; ^6^ College of Veterinary Medicine, Cornell University, Ithaca, NY, United States; ^7^ Department of Biological Sciences, University of Notre Dame, Notre Dame, IN, United States; ^8^ Faculty of Veterinary Medicine, University of Ibadan, Ibadan, Oyo State, Nigeria

**Keywords:** hypoxia, HIFs, NF-kB, immune cells, infectious diseases, inflammation

## Abstract

There are overwhelming reports on the promotional effect of hypoxia on the malignant behavior of various forms of cancer cells. This has been proposed and tested exhaustively in the light of cancer immunotherapy. However, there could be more interesting functions of a hypoxic cellular micro-environment than malignancy. There is a highly intricate crosstalk between hypoxia inducible factor (HIF), a transcriptional factor produced during hypoxia, and nuclear factor kappa B (NF‐κB) which has been well characterized in various immune cell types. This important crosstalk shares common activating and inhibitory stimuli, regulators, and molecular targets. Impaired hydroxylase activity contributes to the activation of HIFs. Inflammatory ligands activate NF-κB activity, which leads to the expression of inflammatory and anti-apoptotic genes. The eventual sequelae of the interaction between these two molecular players in immune cells, either bolstering or abrogating functions, is largely cell-type dependent. Importantly, this holds promise for interesting therapeutic interventions against several infectious diseases, as some HIF agonists have helped prevent immune‐related diseases. Hypoxia and inflammation are common features of infectious diseases. Here, we highlighted the role of this crosstalk in the light of functional immunity against infection and inflammation, with special focus on various innate and adaptive immune cells. Particularly, we discussed the bidirectional effects of this crosstalk in the regulation of immune responses by monocytes/macrophages, dendritic cells, neutrophils, B cells, and T cells. We believe an advanced understanding of the interplay between HIFs and NF-kB could reveal novel therapeutic targets for various infectious diseases with limited treatment options.

## Introduction

**Figure 1:**
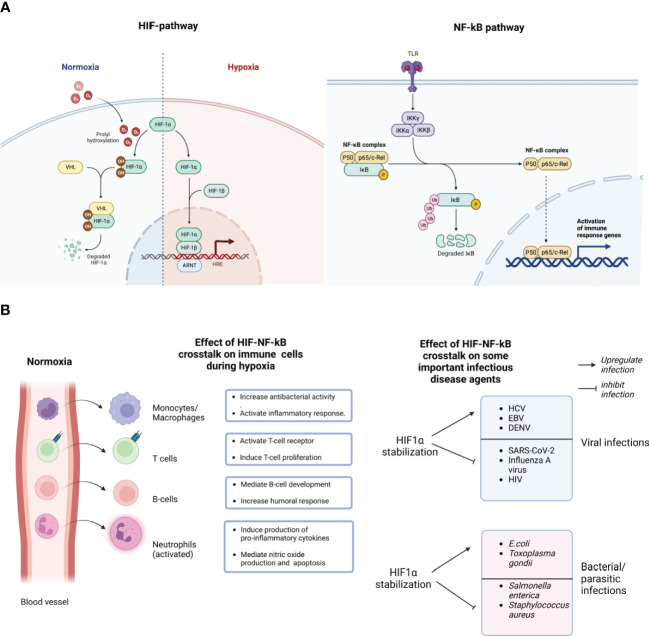
Schematic representation of the molecular crosstalk between the HIF-1a and NF-kB pathways. During normoxia (normal/optimum oxygen tension), prolyl hydroxylase domain (PHDs)-mediated hydroxylation of the prolyl residues of the HIF-1a subunit enables its binding to the von-Hippei-Lindau (VHL) protein, the recognition component of an E3 ubiquitin-protein ligase, resulting in proteasomal degradation. However, during hypoxia, HIF-1α activation requires the enzymatic inhibition of the PHDs-containing proteins. Hypoxia creates an inhibitory effect on PHD-hydroxylation, mediating the stabilization of HIF-1α, subsequent binding of the HIF-1 heterodimer to the promoter regions of HIGs resulting in the hypoxia inducible genes **(A)**. The stabilization of HIF-1α stimulates NF-kB activation by negatively modulating the catalytic activity of the inhibitor of nuclear factor Kappa-b (IKKb). NF-kB has several sub-units like P50, P65 and c-Rel which can be activated by many stimuli, including bacterial lipopolysaccharide (LPS), viral pathogens, cytokines or growth factors, leading to the activation of Toll like receptors (TLR) **(A)**. HIF-1α stabilization enhances the expression of NF-kB-regulated inflammatory cytokines from immune cells like macrophages, neutrophils, T and B cells, after lipopolysaccharides (LPS) stimulation, mediating NF-kB activation of those cells. As shown here, most bone marrow-derived phagocytic cells, essentially granulocytes like neutrophils, monocytes (macrophages in tissues), during peripheral circulation of oxygenated blood in blood vessels, sustain their bactericidal and pro-inflammatory abilities in a "naive state". However, following extravasation and upon invading an infected tissues, they encounter a robust decline in oxygen gradients, and a consequent bolstered activation of their bactericidal and pro-inflammatory abilities ("on-state") **(B)**. Consequently, HIF-1α stabilization could either inhibit the infection and replication of some pathogens while upregulating others. Created with BioRender.com.

Mammalian cells generally adapt to hypoxia (low oxygen tension) by activating a major heterodimeric transcription factor known as hypoxia inducible factor (HIF)-1 (1). HIF-1 is composed of the two main subunits; HIF-1α and HIF-1β. HIF-1α levels are modulated by changes in oxygen partial pressure (pO_2_) within the cellular micro-environment. On the other hand, HIF-1β are constitutively expressed regardless of changes in the extracellular flux (2). HIF-1 regulates the robust expression of hypoxia-inducible genes (HIGs) downstream which are mainly involved in glycolysis (mediated by hexokinase), angiogenesis (mediated by vascular endothelial growth factor), erythropoiesis (mediated by erythropoietin), and cellular proliferation (mediated by adrenomedullin) (3). Mechanistically, HIF-1 activation requires the enzymatic inhibition of the prolyl hydroxylase domain-containing proteins (PHDs), a process which occurs strictly during hypoxia. In a normoxic (normal/optimum oxygen tension) situation, PHDs-mediated hydroxylation of the prolyl residues of the HIF-1α subunit ensures its binding to the von-Hippel-Lindau (VHL) protein, the recognition component of an E3 ubiquitin-protein ligase, leading to an eventual proteasomal degradation (2). However, hypoxia creates an inhibitory effect on PHD-hydroxylation thereby mediating the stabilization of HIF-1α, subsequent binding of the HIF-1 heterodimer to the promoter regions of HIGs resulting in the induction of those genes (4) (**Figure 1A**). Accumulating evidence from literature strongly suggest that HIF-1α plays a key role in the immune response to infection and inflammatory process in mammalian hosts (5–7). Essentially, HIF-1α is critical for the bactericidal activities of phagocytes like macrophages and neutrophils, and vital for inhibiting the systemic dissemination of certain bacteria (8). The role of nuclear factor kappa B (NF-kB) signaling pathway in infectious disease development and control has been well characterized (9, 10). Here, we discussed the interaction between HIF-1α and NF-kB, and their crosstalk within vital components of the innate immune system, highlighting the importance of this crosstalk as a plausible therapeutic target for infectious diseases.

## HIF-1α and NF-kB molecular crosstalk: broad effect on the immune system

The hypoxic response is functionally inter-connected with innate and adaptive immunity (11, 12). Interestingly, the interaction between these stress responses is essentially mediated by a molecular loop regulated by two important transcription factors, HIF-1α and NF-kB (13). Specifically, the stabilization of HIF-1α stimulates NF-kB activation by negatively modulating the catalytic activity of the inhibitor of nuclear factor Kappa-B (IKKb) (14). The hypoxia-induced activation of the NF-kB pathway characterized a while ago (15), shows a direct link between changes in the phosphorylation pattern of I kappa B alpha (IkB-alpha) with NF-kB activation. Following immunoprecipitation of IkB-alpha at different levels of hypoxic exposure, the finding showed an upregulated tyrosine phosphorylation (15). Consequently, the modulation in the phosphorylation of IkB-alpha prevents its degradation and ensures NF-kB binding (15). The ripple effect of this interaction can be seen in various immune cells. For example, bacteria-infected macrophages show a dysfunctional HIF-1α expression following the deletion of the IKKb-encoding gene (14). Also, the role of HIF-1α in enhancing the expression of NF-kB-regulated inflammatory cytokines from macrophages after lipopolysaccharides (LPS) stimulation, and mediating NF-kB activation in anoxic neutrophils has been thoroughly studied (16, 17). Briefly put, most bone marrow-derived phagocytic cells, essentially granulocytes like neutrophils, eosinophils, monocytes (macrophages in tissues), during peripheral circulation in oxygenated blood, sustain their bactericidal and pro-inflammatory abilities in a “naive state”. However, upon invading an infected tissue, they encounter a robust decline in oxygen gradients, and a consequent bolstered activation of their bactericidal and pro-inflammatory abilities (**Figure 1B**) (5). Generally, HIF activity is involved in a series of events promoting the release of proinflammatory cytokines and antimicrobial peptides, enhancing phagocytosis, sustaining phagocytes’ half-life by inhibiting apoptosis, and activating nitric oxide (NO) production (18). NO generally interferes with HIF degradation, causing an amplification loop for phagocyte activation, and the attendant immune response downstream (19). Interestingly, the relatively well-characterized ability of NF-kB to crosstalk with HIF *in vivo* and *in vitro* under inflammatory conditions is still majorly attributed to HIF-1α (5, 6). Very little attention has been given to HIF-1β and HIF-2α subunits in the context of inflammation and infection. Essentially, because both subunits have connections with NF-kB, this could be a premise for future studies. For example, studies evaluating the functional relatedness of HIF‐2α, HIF-1β and HIF‐1α, their roles in the regulation of infection and inflammatory process, and the involvement of other co-stimulatory factors, like pro-inflammatory cytokines, would bolster current molecular understanding of these transcriptional molecules.

## HIF-1α and NF-kB crosstalk: effect on the innate and adaptive immune cells

### Monocytes and macrophages

Macrophages are very important hematopoietic cells derived from circulating monocytes upon migration into tissues and becoming differentiated. These myeloid cells play a vital role both in innate and adaptive immune response (20). They initiate inflammatory responses, phagocytose, and destroy pathogens, and ensure lymphocytes recruitment. They also have the intrinsic ability to show extensive plasticity by undergoing two different types of activation: the classical M1 profile induced by Toll-like receptor (TLR) ligands, inducing a characteristic proinflammatory activity; and the alternative M2 profile, induced by specific interleukins like interleukin (IL)-4 and IL-13), which are largely involved in anti-inflammatory activities (21, 22). During an infection or inflammatory process, a typical host response is monocytosis; infiltration and accumulation of these myeloid cells into the infection and inflammatory site (23). Hypoxia represents an important factor modulating monocytopoiesis, specifically, the maturation from monocytes to macrophages (24). HIF-1*α* and TLRs bidirectionally interact on a plethora of biologically relevant levels (5). While HIF-1*α* regulates the surface expression of various TLRs (e.g., TLR-2, -4, -6, and -9), intracellular signal transduction of several TLRs as aforelisted is often mediated by HIF-1*α*. NF-kB is a major downstream player of this pathway (5). NF-kB remains a major control factor of the mammalian immune system and has been exhaustively reported as being strongly induced by LPS (25). Interestingly, LPS-induced HIF-1α activation is dependent on NF-kB in both human and murine monocytes (26, 27). Functionally, it’s been shown that in response to hypoxia, monocytes switch to a glycolytic metabolism thereby blocking their migration mediated by the chemotactic gradient (28). Gene expression modulations, such as the upregulation of essential molecules needed for macrophage survival, such as glucose transporter 1 (GLUT1) and their association with hypoxia has been well studied as well (29). Conversely, in both human and murine macrophages, hypoxia can initiate gene expression in a HIF-independent manner (14), however, not only through the upregulation of NF-kB, but also activation of transcription factor 4 (ATF4), and early growth response-1 (Egr-1) (30). The strict importance of HIF-1*α* for TLR activation could provide a plausible molecular explanation for the functional importance of HIF-1*α* in many pathogens’ defense.

### Neutrophils

The interaction between NF-kB and neutrophils in a hypoxic cellular milieu is critical in influencing immunological responses and inflammatory processes (5). NF-κB regulates immune-related gene expression, and neutrophils, as major actors in innate immunity, are known for their quick reaction to infections and tissue injury (31). Neutrophils have a short lifespan and are highly motile cells, largely depending on glycolysis as their major energy source (32). As they migrate from the circulation to sites of inflammation, they are required to adapt to and function within lower oxygen tensions because the physiological oxygen gradient is often greatly exaggerated in disease situations. Hypoxia bolsters the association of HIF-1α with the p65 component of NF-kB in neutrophils, hence increasing NF-κB-dependent gene expression (33). Furthermore, the generation of reactive oxygen species (ROS) influences NF-κB activation in neutrophils under hypoxia (34). Hypoxia causes neutrophils to produce more ROS, which then activates NF-κB (34). A study on cancer cells revealed a positive feedback loop between ROS and NF-kB, demonstrating that hypoxia-induced ROS generation activates NF-κB in neutrophils, resulting in the overexpression of pro-inflammatory genes (35). Notably, the connection between NF-κB and neutrophils in a hypoxic milieu is bidirectional since NF-κB activation can impact neutrophil activities in a reciprocal manner (35). Neutrophil migration, survival, and cytokine production have all been demonstrated to be influenced by NF-kB signaling (36). Also, NF-κB activation in neutrophils under hypoxia improves survival by upregulating anti-apoptotic genes (37). HIF-1α-induced activation of NF-κB in neutrophils can also influence the production of cytokines and chemokines, which are important mediators of inflammatory reactions (35).

### Dendritic cells

Dendritic cells (DCs) are vital antigen presenting cells (APCs) between the innate and adaptive immune system (38). Hypoxia and inflammation contribute significantly towards DC differentiation, migration, and survival, essentially mediated by HIF-1α (38). An important activity of DCs is migration to inflammatory sites, often characterized by low oxygen levels. Under this condition, there is an upregulation of HIF-1α levels, and immature myeloid DCs may undergo cell death due to hypoxia (39). However, DC maturation induced by LPS provides protection against hypoxia-induced cell death. This protective activity is mediated by the P13K/AKT pathway, a key intracellular signaling pathway involved in cell growth, motility, survival metabolism, and angiogenesis (39). Importantly, LPS stimulation in a hypoxic environment leads to higher levels of IL-6 and TNF compared to levels produced in normoxia (40). For the induction of TNF in hypoxic conditions, there is a need for HIF-dependent upregulation of MAP3K8 (MAPK kinase kinase 8), an upstream player in the p38/MAPK pathway (39). Conversely, murine experiments with HIF-1α-deficient DCs have shown a decrease in the expressions of IL-12p70, IL-10, TNF, IL-1b, and IL-23 following LPS stimulation in hypoxic bone marrow-derived DCs, strongly suggesting that the induction of these cytokines during hypoxia is independent of HIF-1α in mice (38). HIF-1α has been shown to be vital for DC migration, and the activation of the chemokine receptor CCR7, resulting in an increased glycolysis in humans (39). The elevated glycolysis is required for the rapid, yet transient, migration of DCs to draining lymph nodes, needed for the initiation of protective immune responses and the maintaining homeostasis.

### T- cells

T cells are central players in adaptive immunity (41). In T cell development, NF-kB is a vital factor in the early antigen-independent phase of thymocyte differentiation and the final antigen-dependent lineage commitment and post-selection maturation (42). HIF-1α stabilization in T cells has been shown to occur in response to hypoxia and is essential for maintaining T cell homeostasis and functionality (43). Moreover, HIF-1α stabilization in T cells has been reported to bolster their survival and proliferation, as well as promote the production of effector cytokines, such as interferon-gamma (IFN-γ) and interleukin-17 (IL-17) (43). Furthermore, the interaction between HIFs and T cells in a hypoxic microenvironment influences T cell differentiation (44). HIF-1α has also been implicated in regulating T cell lineage commitment and polarization (44). Specifically, HIF-1α has been shown to promote the differentiation of T helper 17 (Th17) cells, a subset of CD4+ T cells that play a critical role in inflammatory responses and autoimmune diseases (45). The study by Dang et al. demonstrated that HIF-1α directly interacts with the transcription factor RORγt, a master regulator of Th17 cell differentiation, thereby promoting Th17 cell differentiation under hypoxic conditions (46). Moreover, HIF-1α and NF-kB molecular crosstalk can also influence regulatory T cell (Treg) function in a hypoxic microenvironment (47). Tregs are crucial for maintaining immune tolerance and suppressing excessive immune responses. HIF-1α has been found to modulate Treg stability and suppressive capacity (47). This crosstalk promotes the stability and immunosuppressive function of Tregs by upregulating the expression of Foxp3, a key transcription factor involved in Treg development and function (48). Considering the numerous shared activators, inhibitors, and molecular targets between HIF-1α and NF-kB, a deeper insight into their crosstalk in T-cell development and function would greatly advance efforts toward discovering novel targets for T-cell-mediated therapeutic development.

### B cells

B cells are essential components of the adaptive immune system responsible for antibody-mediated immune responses (49). Currently, there is a dearth of information on the effect of the HIF-NF-kB crosstalk in B cells. However, in a hypoxic cellular microenvironment, the interaction between HIF-1 and B cells significantly modulates B cell development, activation, and antibody production (13). Understanding the interplay between HIF-1 and NF-kB in B cells in a hypoxic setting could provide valuable insights into the molecular mechanisms governing B cell responses and immune regulation. An interesting aspect of the interaction between HIF-1 and B cells in a hypoxic microenvironment is the effect of HIF-1 on B cell development and differentiation (12). Hypoxia has been implicated in regulating immunoglobulin production (50). Specifically, HIF-1α has been shown to promote the generation of B cell precursors and enhance their maturation into antibody-secreting plasma cells (50). Also, the molecular interaction between HIF-1α and B cells has been connected to immunoglobulin class switching (51). Moreover, HIF-1α has been shown to regulate the expression of key molecules involved in B cell activation, such as CD40 and CD86 (52).

### HIF-NF-kB crosstalk in inflammatory conditions

The physiological process of inflammation is intricate and involves the activation of multiple coordinated signaling pathways in response to stress (53, 54). Typically, the response entails the expression of small peptides (e.g., cytokines), glycoproteins (e.g., the cluster of differentiation (CD)], and transcription factors, including NF-κB, which contribute to both anti-inflammatory and pro-inflammatory mediators. NF-κB is recognized as the primary pro-inflammatory family of transcription factors and has been identified as a direct modulator of HIF expression in the context of inflammation and hypoxia (53, 54). Generally, chronic inflammatory diseases are characterized by a significant interplay between HIF and NF-κB signaling pathways (53). Hypoxia-induced activation of HIF-dependent genes, along with impaired hydroxylase activity, contribute to the activation of HIF. Inflammatory ligands activate NF-κB activity, which leads to the expression of inflammatory and anti-apoptotic genes (53). For example, mice that overexpress HIF-1α in keratinocytes show enhanced NF-κB activity and elevated expression of pro-inflammatory genes resulting in heightened sensitivity to inflammatory stimuli (55). In contrast, mice lacking HIF-1α in neutrophils revealed the involvement of HIF-dependent NF-κB signaling in the regulation of neutrophil survival. The presence of common target genes and the physical interactions between HIF subunits and NF-κB remains the major reason contributing to their crosstalk in inflammatory settings (4). Studies on several inflammatory conditions, such as rheumatoid arthritis, inflammatory bowel disease, colorectal cancer, asthma, and many others, show the presence of potential points and the similarity in the cellular microenvironment in those conditions, which underscore the importance of the crosstalk between HIF and NF-κB (55–57).

### HIF-NF-kB crosstalk in infectious disease conditions

There is growing evidence that the crosstalk between these two pathways may be critical in the pathogenesis of a range of infectious diseases, including viral, bacterial, and parasitic infections (58). **Figure 1B** provides a short list of viral, bacterial and parasitic infectious diseases that are impacted by hypoxia and consequently could be modulated, either inhibition or upregulation, by the molecular crosstalk between HIFs and NF-kB. Essentially, as described above, the hypoxic response is crucial for the optimum activities of tissue macrophages and infiltrating neutrophils that encounter low oxygen pressure in infected tissues. Also, HIF-1α has been proposed to bolster the expression of inflammatory cytokines that are known to be regulated by NF-κB, particularly in LPS-stimulated macrophages (59). It has been suggested that the activation of HIF-1α during Infection is particularly attributed to common mechanisms associated with the Infection, such as a hypoxic environment caused by bacterial oxygen consumption (59). The role of hypoxia on infectious agents is bidirectional. Several studies have shown the activation of HIF-1α in response to infections caused by many pathogens either upregulate or downregulates their replication in host cells (60, 61). On the other hand, it has been suggested that the presence of bacteria can also stabilize HIF-1α in both immune cells and epithelial cells (60). Upon stabilization and activation, HIF-1α initiates the release of anti-microbial peptides like cathelicidins and granule proteases, which facilitate the production of TNF- α and nitric oxide (62). Considering a highly debilitating infectious disease, tuberculosis caused by *Mycobacterium tuberculosis* (Mtb), NF-κB is involved in the transcriptional activation of HIF-1 (61). Studies have observed a higher expression of HIF-1α and related immune responses in Mtb infection both *in vivo* and *in vitro* (63, 64). When murine macrophages are infected with Mtb, inducible NO synthase (NOS2/iNOS) increases nitric oxide (NO), leading to the inhibition of PHDs and creating a positive feedback loop that results in sustained high levels of HIF-1α and increased macrophage activation (61). Through the regulation of HIF-1α activation in macrophages during microbial infections, which can result in decreased oxygen levels, NF-κB has the ability to boost glycolytic energy metabolism, the production of angiogenic factors, and the expression of pro-inflammatory cytokines, chemokines, and anti-microbial peptides. As a result, the ability of NF-κB to enhance the expression of HIF-1α broadens its regulatory capabilities, leading to the more effective execution of the host-defense response. Furthermore, modulations in oxygen tension and attendant HIF-1α signaling may also play a vital role in viral tropism and pathogenesis (65, 66). Therefore, pharmaceutical agents that modulate the HIFs pathway could provide novel treatment options for infections and associated pathological conditions. However, advanced investigations are required to decipher the complex interactions between different pathogen-specific genes and the crosstalk between HIFs and NF-kB. One aspect of advanced investigations involves unraveling the intricate interplay between HIFs and pathogen-specific genes. Different pathogens have evolved diverse mechanisms to interact with host cells and manipulate cellular processes. Understanding how HIFs modulate the expression of pathogen-specific genes is crucial for deciphering the underlying molecular mechanisms of pathogenesis and identifying potential therapeutic targets. This requires comprehensive studies using advanced genomics, and transcriptomics approaches to characterize the transcriptional changes induced by HIFs during Infection (66, 67).

Furthermore, investigating the crosstalk between HIFs and NF-kB is of paramount importance. Both HIFs and NF-kB are central players in inflammation and immune responses, and their interactions can significantly influence the outcome of infectious diseases. Deciphering the molecular mechanisms underlying their cross-regulation is essential to uncover the complex dynamics of host-pathogen interactions. Advanced investigations, including molecular and cellular studies, can shed light on the reciprocal regulation between HIFs and NF-kB, enabling the development of therapeutic strategies that target both pathways synergistically (68, 69). In addition, advanced investigations should explore the functional consequences of modulating HIFs on host immune responses. HIFs can modulate immune cell function, cytokine production, and anti-microbial responses, thereby influencing the outcome of infections. Elucidating the impact of HIFs modulation on immune cell behavior, including phagocytosis, antigen presentation, and T cell responses, is critical for understanding the immunological consequences of targeting the HIFs pathway. This can be achieved through advanced immunological techniques, such as flow cytometry, functional assays, and *in vivo* models (70, 71). Moreover, advanced investigations should incorporate systems biology approaches to gain a comprehensive understanding of the complex molecular networks involved in infectious diseases. Integrating multi-omics data, including genomics, transcriptomics, proteomics, and metabolomics, can provide a holistic view of the host-pathogen interactions including signaling cascades for different pathogens. These research along with computational analyses can unravel the intricate molecular pathways and identify key nodes or modules that can be targeted for therapeutic intervention (72, 73).

## Concluding remarks and perspectives: HIF-NF-kB crosstalk as potential therapeutic targets

Developing pharmaceutical HIF-1α agonists to inhibit the microbial activity of pathogens in different cell types could be an exciting strategy for supplementary therapy against complex infections caused by antibiotic-resistant pathogens or treating infections in patients with compromised host immunity. As aforementioned, HIF-1α is increasingly being investigated as a potential target in the fight against bacterial, viral and parasitic infections, particularly multidrug-resistant ones. Modulating HIF-1α as a pharmacologic intervention for treating chronic inflammatory disorders or improving innate immune function has been exhaustively proposed (58). Moreover, inhibiting HIF-1α activity may be a promising therapeutic approach for sepsis triggered by LPS (74). To develop effective therapeutic strategies for infectious and inflammatory diseases, a solid grasp and understanding of the underlying biology is required (75). First, there is a need to identify novel genes and targets specific to various pathogens which can be modulated directly or indirectly by HIF-1α inhibition. **Table 1A** provides a list of genes that have been tested for various pathogens in the context of HIF-NF-kB crosstalk and therapeutic development. Moreover, in general terms, comprehensive research efforts are required to uncover specific molecules, receptors, and signaling pathways that play crucial roles in the establishment of Infection and evasion of host immune responses. Identifying these novel targets would enable the design of more precise and targeted therapeutic interventions. Second, an important point for consideration in therapeutic design is usually having a grasp of the dynamics of the pathogen’s variability. Infectious agents exhibit genetic and phenotypic variability, which can influence their virulence, drug resistance, and ability to evade host immune responses (75). Therefore, investigating the molecular mechanisms underlying pathogen variability and its impact on disease progression and therapeutic outcomes could be germane in this context. This includes studying genetic diversity, mutations, and genetic exchanges among pathogens, as well as characterizing their phenotypic changes and adaptation to different host environments (either in normoxia and hypoxic cellular environment). Additionally, as mentioned above, the integration of multi-omics approaches is necessary to gain a comprehensive understanding of the molecular pathways involved in infectious diseases. Utilizing genomics, transcriptomics, proteomics, and metabolomics can provide insights into the complex interactions between host and pathogen (75). Data garnered from these advanced approaches, together with computational modeling and network analysis could provide a robust understanding of the effect of the HIF-NF-kB crosstalk on infectious disease processes. This integrative approach is vital for obtaining a holistic view of the underlying biology and identifying novel therapeutic targets. Lastly, it is crucial to validate findings from preclinical studies and translate them into clinical applications. While *in vitro* and animal studies provide valuable insights, it is essential to validate the efficacy and safety of potential therapeutic strategies in human subjects. **Table 1B** shows a list of current HIF-1α inhibitory or upregulating molecules and drugs currently under clinical trials. These studies would ideally involve conducting well-designed clinical trials to assess the therapeutic potential of identified targets. For this, physiologically relevant disease models can be employed to answer the needed research questions. In this regard, novel animal models such as the zebrafish, in combination with traditional cell lines and murine models, could help to elucidate the extent of the effects of HIF modulation on cellular physiology (157). We strongly believe targeting HIF-NF-kB crosstalk holds promise for therapeutic interventions for various microbial infections. Studies employing genetic and chemical inhibition of the PHD proteins have demonstrated therapeutic benefits in various disease models, such as colitis, where inflammation and NF-kB activity are significant contributors (158). Therefore, it would be intriguing to investigate the potential therapeutic impact of HIF activators and inhibitors on other infectious agents. More research efforts are needed to test the efficacy of available HIF modulators in different disease models, which could provide valuable insights into the various forms of HIF-NF-kB crosstalk that occur in different cell types in the context of individual pathogen. Collectively, a progressive understanding of the interplay between HIF-1 and NF-κB pathways through hydroxylation (159) can lead to the development of novel therapies for infectious and inflammatory disorders.

**Table 1A:** Table showing the effects of hypoxia on genes of various infectious agents.

Pathogens	Genes	Hypoxia induced impact	References
Bacteria			
Pseudomonas aeruginosa	AQ quorum sensing signaling molecules and PQS	2-alkyl-4-quinolone (AQ) quorum sensing (QS) signaling molecules and pseudomonas quinolone signal (PQS) degrades HIF-1 leading to HIF-1α downregulation in *P. aeruginosa* pathogenesis	Legendre et al., (76)
Pseudomonas aeruginosa	AtvR	Nitrate-induced hypoxia causes expression of *atvR* and consequently increase *P. aeruginosa* growth due to potentiated expression of nitrate reductase genes	Kaihami et al., (77)
Pseudomonas aeruginosa	AdhA	Anr transcription factor activates the a*dhA* gene in *P. aeruginosa* as a response to ethanol-induced hypoxia.	Crocker et al., (78)
Pseudomonas aeruginosa	PPHD	Pseudomonas prolyl hydroxylase (PPHD) serves as oxygen sensing enzyme in *P. aeruginosa* and it hydroxylates HIF1α subunit in presence of sufficient oxygen whereas in limited oxygen, HIFα accumulates and translocates to the nucleus where it transcriptionally activates genes involved in erythropoiesis and angiogenesis. These activities thereby affect the pathogenicity and antibiotic resistance of *P. aeruginosa*	Schaible et al., (79)
Coxiella burnetii	CBP	*C. burnetii* infection promotes HIF1α-mediated upregulation of several metabolic target genes, like *C. burnetii* protein (CBP), and enhances apoptosis-regulators towards a more pro-apoptotic signature. Under hypoxic conditions, inflammatory genes are shifted towards a pro-inflammatory cytokine profile.	Hayek et al., (80)
Bartonella henselae	BadA (type IV pilli)	HIF1 stabilized and activated in *Bartonella* infections causes and promotes secretion of proangiogenic cytokines in humans and rodents	Riess et al., (81); Pasipoularides et al., (82)
Mycobacterium tuberculosis	DosR regulon	The DosR regulon enables pathogen to persist during lengthy hypoxia by regulating genes such the genes involved in alternative electron transport pathways (*fdxA*), nitrate metabolism (*narK2* and *narX*), triglyceride synthetase (*tgs1*), and deoxynucleoside triphosphate synthesis under microaerophilic conditions (*nrdZ*).	Chen et al., (83).
Mycobacterium tuberculosis	Rv0081	Hypoxia regulates Rv0081 activities by promoting interaction with the DosR regulon.	Galagan et al., (84)
Mycobacterium tuberculosis	Clp gene regulator (Rv2745c)	Clp protease induction activates the *clgR* and reverse the hypoxic condition	McGillivray et al., (85),
Mycobacterium tuberculosis	TreS	Tres is upregulated and associated with hypoxia-induced metabolic reprograming of *M. tb*	Eoh et al., (86)
Mycobacterium tuberculosis	Rv0998	Helps in adapting to hypoxia by acetylating DosR (negative influence) potentiating *M. tb* pathogenesis	Tai et al., (87)
Mycobacterium tuberculosis	MtrB	MtrB interacts with DosR (a noncognate RR) in a phosphorylation-independent manner during hypoxic conditions to establish infection caused by *M. tuberculosis*	Gutierrez-Rodarte et al., (88)
Salmonella enterica	Sal (Siderophore)	Enhance VEGF secretion following HIF-1α activation	Kostic et al., (89)
Yersina enterocolitica	Ybt (Siderophore)	Upregulated following HIF-1α activation, potentiating infection and replication of *Yersina enterocolitica*	Kostic et al., (89)
Staphylococcus aureus	SrrAB two-component system	Gene resistance to hypoxia, inhibiting hypoxic induced impact on Staphylococcus aureus infection	Kinkel et al., (90)
Viral			
Dengue virus	Vascular endothelial growth factor (VEGF)	Hypoxic condition leads to the binding of the hypoxia-responsive element (HRE) in the promoter of the VEGF thereby controlling the transcription and expression of the VEGF.	Mottet et al., (91) Frakolaki et al., (92)
Human immunodeficiency virus	VPR	Hypoxia significantly increases glucose uptake in adipose−derived stem cells (ASCs), and glucose transporter (GLUTs). HIF-1α interacts with HIV-1 accessory protein viral protein R (Vpr) thereby inducing HIV gene expression Hypoxia decreases CDK9/cyclin T1 and Sp1 expression, producing a reduction in HIV Tat expression.	Charles et al., (93); Deshmane et al., (94)
Vesicular stomatitis virus	eIF2α	Hypoxia decreases VSV mRNA at early time points after infection of HeLa cells, but at later times after infection, VSV reduces eIF2α phosphorylation, promoting viral protein synthesis which results in VSV replication	Connor et al., (95)
SARS-CoV-2	ACE2	HIF-1α downregulates the expression of ACE2 and TMPRSS2 SARS-CoV-2 receptors. HIF-1α induces Angiotensin II, which inhibits ACE2 synthesis which results in SARS-CoV-2 replication	Zhang et al., (96) Wing et al., (66)
Epstein–Barr virus (EBV)	EBV transcription factor Zta	Hypoxia increases Zta expression thus increase replication of EBV. HIF-1α interacts with the latent-lytic switch BZLF1 gene Zp in HEK 293T cells thus increase the lytic gene expression	Jiang et al., (97); Kraus et al., (98)
Herpes simplex virus type 1 (HSV-1) mutant G207	GADD34	Hypoxia increases the expression of GADD34 in the U87 human glioma cells which leads to enhance the replication of HSV-1	Aghi et al., (99)
Adenovirus	EA1	Hypoxia decreases the EA1 protein expression which leads to the reduction in adenovirus replication *in vitro*	Shen and Hermiston, (100)
Parasitic			
Leishmania donovani	J774 cells	HIF-1α stability promotes LD infection and vice versa.	Singh et al., (101)
Trypanosoma cruzi	Epimastigotes	An increase in proliferation and a reduction in metacyclogenesis Parasites cultured in hypoxia produced more reactive oxygen species (ROS) Hypoxia trigger a shift in the bioenergetic metabolism of T. cruzi epimastigotes, favoring ROS production and fermentation to sustain ATP production, allowing the parasite to survive and proliferate in the insect vector	Saraiva et al., (102)
Clonorchis sinensis	*CsMb (CsMb1—CsMb3), CsNgb*, and *CsGbX*,	Hypoxia potentiates the expression of these *Clonorchis* genes, named *CsMb* (*CsMb1—CsMb3*), *CsNgb*, and *CsGbX*, according to their preferential similarity patterns toward respective globin subfamilies, exponentially increased in the worms coinciding with their sexual maturation, after being downregulated in early juveniles compared to those in metacercariae.	Saraiva et al., (102)
Toxoplasma gondii	Toxoplasma strain RH	HIF-1α stability potentiates Toxoplasma infection and vice versa by preventing HIF-1α prolyl hydroxylation. Infection significantly decreases PHD2 abundance, which is the key prolyl hydroxylase for regulating HIF-1 α.	Wiley et al., (103)
Plasmodium	Human hepatocytes	Ambient hypoxia increases liver-stage malaria infection *in vitro* Hypoxia however does not increase sporozoite-dependent or host-dependent invasion	Ng et al., (104)

**Table 1B:** Table showing a list of HIF-NF-kB modulators highlighting their mechanism of action, accompany Clinical trial numbers (NTC), and related pathogen/diseases.

Modulators	List of drugs	Mechanisms	Success	Related Diseases/Infectious Pathogens	Clinical trials number (NCT)	References
HIF	PI3Kinase inhibitors: Wortmannin	Reduce HIF-1α protein levels and mRNA accumulation.	Improve the quality of life of patients with multiple sclerosis	Relapsing remitting multiple sclerosis	NCT00534261	Blommaart et. al. (98), Jiang et al. (105)
	LY294002	Reduce HIF-1α protein levels and mRNA accumulation	Same as Wortmannin, but more stable in solution, and inhibits formation of autophagosome	Breast cancer	NCT02337309	Caffa et al. (106), Jiang et al. (105)
	mTOR inhibitor: Rapamycin	Reduce HIF-1α protein levels and mRNA accumulation. Also inhibits interleukin-2-induced phosphorylation,and activation of p70 S6 kinase	Enhance chondrongenic differentiation Potent immunosuppressant	Lymphatic malformations MERS-CoV infection (re-purposed)	NCT03243019	Majumder et al. (107) Preitschopf et. al (108)., Patocka et. al (109).,
	Cardiac glycosides such as Digoxin, Ouabain	Inhibit HIF-1α protein expression and decrease growth of tumor xenografts. Inhibit viral RNA synthesis.	Generally used for patients with heart diseases, but can be repurposed for antiviral functions	Heart failure DNA and RNA viruses, such as cytomegalovirus, herpes simplex virus, MERS-CoV, HIV, respiratory syncytial virus, Chikungunya virus SARS-CoV-2 (re-purposed)	NCT02797145	Semenza (110) Cho et. al (111).,
	Microtubule targeting agents such as 2-Methoxyestradiol (2ME2)	Prevent HIF-1α translation and nuclear accumulation. Suppress immune cell activation	Undergoing clinical trials in patients with breast, prostate, and ovarian cancer.	Refractory multiple myeloma Autoimmune encephalomyelitis	NCT00028821	Mabjeesh et al. (112) Xia et al. (113) Duncan et. al (114).,
	Class II histone deacetylase (HDAC) inhibitors such as trichostatin and LAQ824	Increase HIF-1α ubiquitination and degradation of HIF-1α by regulating hepcidin Inhibit viral replication	Chromatin remodeling and regulation of gene expressions	HCV, HIV, RSV	Not available	Herbein et. al (115).,
	EZN-2968	Inhibition of HIF-1α and HIF-1α-dependent genes	Completed study on the ability to reduce growth in xenografts.	Hepatic metastasis	NCT01120288	Greenberger et al. (116)
	Ibuprofen and other NSAIDs	Decrease HIF-1α and HIF-2α protein level. Probable upregulation of ACE-2	Withdrawn from clinical trials	Coronaviruses	NCT04383899	Palayoor et al. (117)
	Geldanamycin (GA)	Prevent binding of Hsp90 to HIF-1α thereby decreasing its stability with subsequent proteasomal degradation. Also inhibits viral replication.	Now in Phase II clinical trials in patients with VHL disease, breast cancer, etc	Advanced malignancies HSV-2	NCT00003969	Isaacs et al. (118) Semenza (119), Li et. al (120).,
	Radicicol	Prevent binding of Hsp90 to HIF-1α thereby decreasing its stability with subsequent proteasomal degradation. Targets Nonstructural proteins of flaviviruses	Suppresses Chikungunya virus (CHIKV) replication by blocking the synthesis of both positive- and negative-strand viral RNA as well as expression of viral proteins.	CHIKV	Not available	Isaacs et al. (118) Nam et. al (121).,
	Antimycin A	Induces apoptosis and inhibits the mitochondrial electron transport chain from cytochrome b to cytochrome C1, decreased HIF-1α protein level by an unknown mechanism	Broad spectrum antiviral activity	*Members in the families Togaviridae*, *Flaviviridae*, *Bunyaviridae*, *Picornaviridae*, and *Paramyxoviridae*	Not available	Maeda et al. (122)
	Moracin O and P	Increase HIF-1α degradation by activating proteasomal system or by unknown mechanisms.	Broad spectrum anti-inflammatory agent.	Not available	Not available	Dat et al. (123) Hardianti et. al (124).,
	Manassantin B	Degradation of HIF-1α and inhibition of VEGF secretion.	Inhibits Coxsackievirus B3 (CVB3) replication.	CVB3	Not available	Hossain et al. (125) Song et. al (126).,
	Curcumin and berberine	Increases HIF-1α proteasomal degradation.	Ongoing trial	Diverticulitis	NCT05596214	Choi et al. (127)
	Resveratrol	Increases HIF-1α proteasomal degradation.	Completed study. Reduce pain due to endometriosis	Endometriosis	NCT02475564	Park et al. (128) Dull et. al (129).,
	Methylalpinumisoflavone	Increases HIF-1α proteasomal degradation.	Unknown status	*Streptococcus spp*	Not available	Liu et al. (130) Lim et. al (131).,
	Sibiriquinone A	Suppresses HIF-1α accumulation and VEGF secretion through HIF-1α degradation	Potent anti-inflammatory compound	Not available	Not available	Dat et al. (132)
	Acriflavine	Binds to the PAS-B subdomain of HIF1a and HIF-2α thereby preventing the binding to HIF1b, an effect that results in reduced VEGF production and tumor growth.	Broad antiviral activity against SARS-CoV-2 and other betacoronaviruses at nanomolar concentration	SARS-CoV-2 and other betacoronaviruses	Not available	Lee et al. (133) Napolitano et. al (134).,
	Korean red ginseng	Inhibit HIF-1α and 1b dimerization with no toxic effects	On-going trial	Depression	NCT01496248	Choi et al. (135)
	Echinomycin	Binds to DNA and inhibits HIF1α activity	Unknown	Tuberculosis *Streptomyces spp*	Not available	Wang et al. (136)
	Doxorubicin and daunorubicin	Bind to DNA and prevent HIF binding, transcription of target genes, and tumor growth.	Completed study. Complete remission following treatment.	Adult acute lymphoblastic leukemia	NCT03419494	Tanaka et al. (137) Chen and Gaber (12),
	Chetomin	Prevents HIF-p300 binding by acting on p300 structure and inhibit transcription of HIF target genes.	Inhibits SARS-CoV-2 3C-like Protease (3CL^pro^)	SARS-CoV-2	Not available	Kung et al. (138) Ibrahim et. al (139).,
	Bortezomib	Binds to the domain of HIF1α that interacts with p300 thereby preventing a functional interaction between these two factors and blocking transcription of target genes.	Approved in the US by FDA for use in multiple myeloma, based on the results from the Phase II trial. Two open-label, Phase III trials established the efficacy of bortezomib 1.3mg/m^2^ (with or without dexamethasone) in patients with relapsed/refractory multiple myeloma.	Relapsed/refractory multiple myeloma	NCT02976272	Befani et al. (140)
NF-kB	B-carboline	Prevents the activation of IKK kinases.	Suppress viral infection by interfering with the viral replication of the influenza A/H5N1 virus.	Human and Avian influenza	Not available	Karin et al. (141) Hegazy et. al (142).,
	Parthenolide	Prevents the activation of IKK kinases.	Completed study. Resolves skin irritation.	Allergic contact dermatitis	NCT00133341	Gupta et al. (143)
	Curcumin	Prevents the activation of IKK kinases.	Completed study. Beneficial for patient with advanced pancreatitis and postprandial inflammation with modulation in blood levels of anti- and pro-inflammatory markers	Postprandial Inflammation in Men and Postmenopausal Women	NCT01964846	Lubbad et al. (144)
	Dehydroxymethylepoxyquinomicin (DHMEQ)	Inhibits NF-kB nuclear accumulation with anti-inflammatory and anti-tumoral activity.	Unknown	Liver Fluke Associated Cholangiocarcinoma	Not available	Kozakai et al. (145) Seubwai et. al (146).,
	Kinase inhibitors such as SB203580, PD0980589, tyrosine kinase inhibitors, betaine, etc.	Prevents the activation of IKK kinases.	Potential antiviral activity	Coronaviruses (re-purposed)	Not available	Vanden Berghe et al. (147) Naik et. al., (148)
	Proteasome inhibitor such as Bortezomib, ALLnL, lactacystine, MG132	Inhibition of IkB degradation by the proteasomal system.	Minimal side effects following inhibition of NF-kB	SARS-CoV-2 (repurposed)	Not available	Wilczynski et al. (149)
	Second-generation proteasome inhibitors such as carfilzomib and salinosporamide	Inhibition of IkB degradation by the proteasomal system.	Have a lower toxicity and can be delivered orally	*Plasmodium* and *Mycobacterium tuberculosis*	Not available	Wilczynski et al. (149); Kale and Moore (150) Ignatz-Hoover et. al (151)
	Sirtuins	Inhibit NF-kB acetylation and consequent activation.	Unknown status. Potential antiviral functions.	COVID-19 HBV	NCT04907916	Chen and Greene (152) Kong et. al (153)
	Acetyltransferases such as p300 and CREB-binding protein	Inhibit NF-kB acetylation and consequent activation.	Ongoing trial	Rubinstein-Taybi syndrome	NCT04122742	Chen and Greene (152)
	Gallic acid	Inhibit NF-kB acetylation and consequent activation.	Unknown status	Dyslipidemia	NCT03805139	Choi et al. (154)
	SN50	Preventing NF-kB nuclear translocation	Unknown status	*Neisseria meningitidis*	Not available	Griffiths et. al., (155) Sun et al. (156)

## Data availability statement

The original contributions presented in the study are included in the article/supplementary material. Further inquiries can be directed to the corresponding author.

## Author contributions

Conceptualization: OCA. Figure and Table: OCA, SS, SCO, RA, ROA. Article draft and review: All authors contributed to the writing and review of the manuscript.
